# Correlating Mass Spectrometry Imaging and Liquid Chromatography-Tandem Mass Spectrometry for Tissue-Based Pharmacokinetic Studies

**DOI:** 10.3390/metabo12030261

**Published:** 2022-03-18

**Authors:** Andreas Dannhorn, Emine Kazanc, Gregory Hamm, John G. Swales, Nicole Strittmatter, Gareth Maglennon, Richard J. A. Goodwin, Zoltan Takats

**Affiliations:** 1 Department of Metabolism, Digestion and Reproduction, Imperial College London, London SW7 2AZ, UK; andreas.dannhorn1@astrazeneca.com (A.D.); e.kazanc17@imperial.ac.uk (E.K.); 2 Imaging & Data Analytics, Clinical Pharmacology and Safety Sciences, R&D, AstraZeneca, Cambridge CB4 0WG, UK; gregory.hamm@astrazeneca.com (G.H.); john.swales@astrazeneca.com (J.G.S.); nicole.strittmatter@tum.de (N.S.); richard.goodwin@astrazeneca.com (R.J.A.G.); 3 Oncology Safety, Clinical Pharmacology and Safety Sciences, R&D, AstraZeneca, Cambridge CB4 0WG, UK; garethadam.maglennon@astrazeneca.com; 4 Institute of Infection, Immunity and Inflammation, College of Medical, Veterinary and Life Sciences, University of Glasgow, Glasgow G12 8TA, UK; 5 Laboratoire PRISM, Inserm U1192, University of Lille, Villeneuve d’Ascq, 59655 Lille, France; 6 The Rosalind Franklin Institute, Harwell OX11 0QG, UK

**Keywords:** DESI, MALDI, mass spectrometry imaging, DMPK, drug absorption, tissue imaging

## Abstract

Liquid chromatography-tandem mass spectrometry (LC-MS/MS) is a standard tool used for absolute quantification of drugs in pharmacokinetic (PK) studies. However, all spatial information is lost during the extraction and elucidation of a drugs biodistribution within the tissue is impossible. In the study presented here we used a sample embedding protocol optimized for mass spectrometry imaging (MSI) to prepare up to 15 rat intestine specimens at once. Desorption electrospray ionization (DESI) and matrix assisted laser desorption/ionization (MALDI) mass spectrometry imaging (MSI) were employed to determine the distributions and relative abundances of four benchmarking compounds in the intestinal segments. High resolution MALDI-MSI experiments performed at 10 µm spatial resolution allowed to determine the drug distribution in the different intestinal histological compartments to determine the absorbed and tissue bound fractions of the drugs. The low tissue bound drug fractions, which were determined to account for 56–66% of the total drug, highlight the importance to understand the spatial distribution of drugs within the histological compartments of a given tissue to rationalize concentration differences found in PK studies. The mean drug abundances of four benchmark compounds determined by MSI were correlated with the absolute drug concentrations. Linear regression resulted in coefficients of determination (R^2^) ranging from 0.532 to 0.926 for MALDI-MSI and R^2^ values ranging from 0.585 to 0.945 for DESI-MSI, validating a quantitative relation of the imaging data. The good correlation of the absolute tissue concentrations of the benchmark compounds and the MSI data provides a bases for relative quantification of compounds within and between tissues, without normalization to an isotopically labelled standard, provided that the compared tissues have inherently similar ion suppression effects.

## 1. Introduction

Quantification of drugs present in tissue samples by liquid chromatography followed by tandem mass spectrometry (LC-MS/MS) is a standard tool in pharmacokinetics and drug distribution studies [1,2,3]. The ability to use standardized sample preparation and analysis protocols make liquid chromatography-tandem mass spectrometry (LC-MS/MS) analysis quantitative and reproducible. However, the results always represent the average concentration values for the entire analyzed specimen and all spatial information is lost in the tissue homogenization step during sample preparation. Mass spectrometry imaging (MSI) allows qualitative and quantitative, multiplexed detection of xenobiotics [4,5,6,7,8] and endogenous small metabolites, lipids and peptides present in tissue sections [9,10]. Recent studies demonstrate near cellular resolution analysis allowing to resolve small morphological tissue features [11,12,13]. The achievable high spatial resolution enables to follow the distribution of endogenous metabolites and xenobiotics into small morphological tissue compartments. The impact of high spatial resolution imaging could be demonstrated in studies evaluating blood-brain barrier permeation in rodents [14] or studying the distribution of endogenous metabolites and xenobiotics along the villi-crypt axis in rat intestines [15]. Laser capture microdissection (LCM) in conjunction with LC-MS/MS quantification of drugs in the dissected tissues has been described as an alternative technique leveraging the sensitivity and robustness of LC-MS/MS based drug quantification whilst adding a spatial dimension to the data. However, whilst literature reports indicate higher sensitivity compared to MALDI-MSI [16], LCM-LC-MS/MS has not found widespread use for the determination of drug distributions and is preferentially used to evaluate changes in the proteome or transcriptome of tissues in the context of drug treatment [17,18]. The low uptake of the LCM approach is likely based on the complexity of the protocols and the need for specialized equipment.

Absolute quantification is achieved in MS experiments by normalizing the abundance of a given analyte to the abundance of an isotopically labelled standard with known concentration and use of a typically linear calibration function to derive the concentration value. For such experiments, the isotopically labelled standard needs to be homogenously distributed inside the sample. Since it is not possible for solid samples, the best approximation for MALDI-MSI experiments is to mix the standard into the matrix solution and spray-deposit on the samples [7]. Analogously, spray-deposition of labelled standards has been used for the absolute quantification in DESI-MSI experiments [19]. Translation of the normalized abundances is most commonly achieved through integration against tissue homogenates spiked with the analyte of interest [4] or disposition of a dilution series onto control tissue sections [5]. In contrast, relative quantitation is often achieved by comparing the relative abundances of a compound in different tissues or histological compartments. When performing relative quantification of analytes across various tissues, the use of a homogenously deposited labelled standard seems advisable as ion suppression effects can vary considerably between tissues [20]. As the use of isotopically labelled standards cannot be evenly distributed inside tissue samples for the absolute quantification of endogenous metabolites or drugs during the early development phases, relative quantification if often performed without the use of any internal standard to compensate for local ion suppression effects. In the present study we aimed to compare the results of LC-MS/MS quantification and MSI to study the distribution and absorption of four orally co-administered drugs (terfenadine, losartan, dextromethorphan and diphenhydramine) in the rat intestine. To facilitate the sample preparation, we applied our published sample embedding and preparation protocol [21] to prepare 15 intestine specimens for MSI analysis simultaneously. The spatial distributions of the cassette-dosed drugs within the specimens were determined by desorption electrospray ionization-(DESI) and matrix-assisted laser desorption/ionization-(MALDI) MSI. To evaluate the quantitative relation of the MSI data, the mean drug abundances, determined by MSI, were correlated with the absolute concentrations determined by LC-MS/MS analysis. In contrast to LC-MS/MS based quantification, high resolution MSI, performed at 10 µm spatial resolution, offered the ability to precisely localize the drug disposition within the intestinal morphological features and to precisely determine location and rate of drug absorption. The additional information about the spatial distribution of drugs within the specimen could also be leveraged to differentiate between the absorbed drug fraction and the residue remaining in the intestinal lumen.

## 2. Results

**Figure 1:**
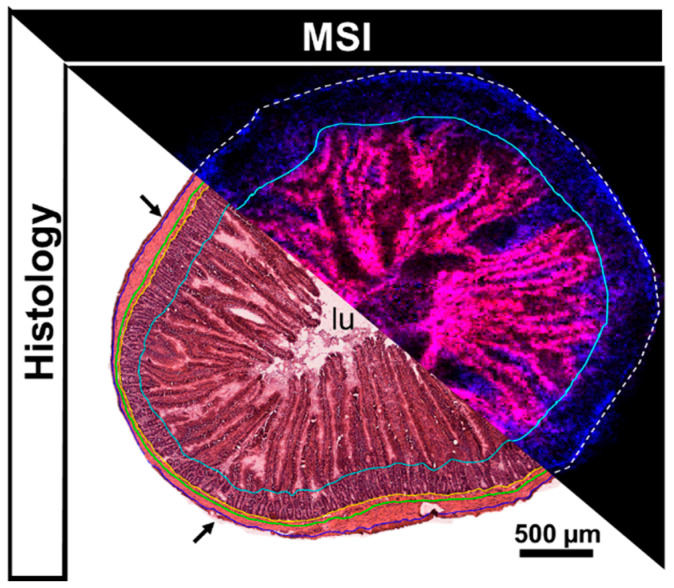
Annotated H&E scan compared to combined ion image obtained from an adjacent tissue section analyzed by MALDI-MSI (endogenous lipids PC (34:2) [M+K]^+^ (*m*/*z* 796.53) (red) and PC (34:1) [M+K]^+^ (*m*/*z* 798.54) (blue). Histological annotation on the H&E stained tissue: arrows = serosa, outside blue line = outer muscularis, between blue and green line = inner muscularis, between green and yellow line = submucosa, between yellow and turquoise line = mucosal crypts, within turquoise line = mucosal villi and intestinal lumen (lu).

The intestine is a complex organ consisting of multiple morphological compartments such as the muscularis, submucosa, the mucosa with the mucosal crypts and villi as well as the intestinal lumen (Figure 1). Whilst high resolution MSI allows to determine the spatial distribution of analytes and in correlation with small morphological features such as mucosal villi, homogenization and extraction of the drugs for LC-MS/MS analysis destroy the tissue morphology and retrospective linkage of the determined concentrations to distinct compartments is impossible.

**Figure 2:**
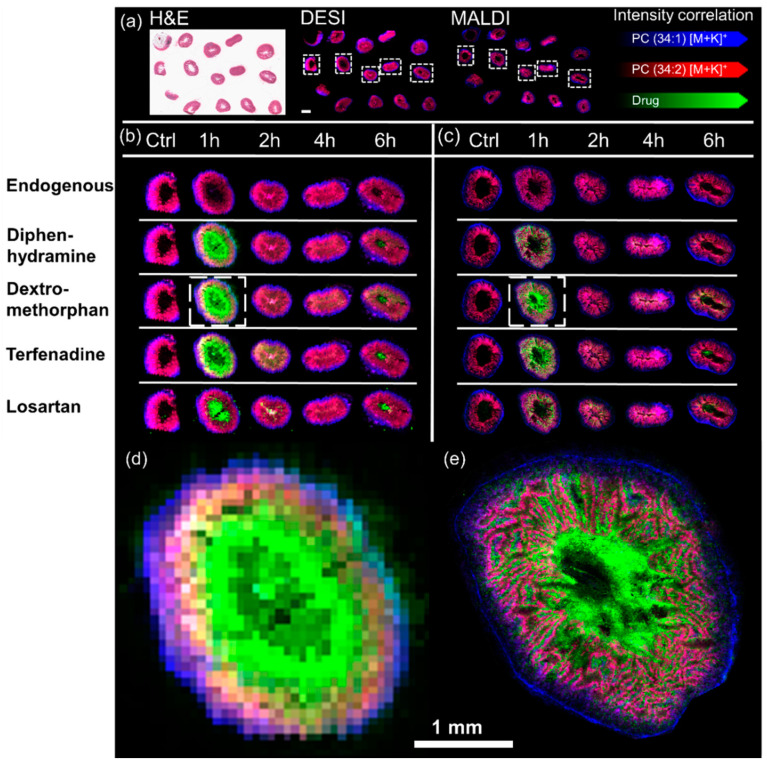
Representative spatial distribution of the cassette dosed drugs determined by DESI-MSI and MALDI-MSI: (**a**) Panorama view on a whole post-DESI H&E stained slide (left) as it was analyzed in one experiment by DESI-MSI (middle) and MALDI-MSI (right). The scale bar in the DESI insert is 2 mm wide. The spatial distribution of all four dosed drugs is shown in one representative replicate for all timepoints in relation to endogenous species outlining the tissues. The left-hand side of the figure shows the results of (**b**) DESI-MSI experiments performed with a spatial resolution of 75 µm whilst the right-hand side of the figure shows the results of (**c**) MADI-MSI experiments acquired with a spatial resolution of 10 µm. The inserts (**d**,**e**) display the spatial distribution of dextromethorphan in the highlighted tissue sections in (**b**,**c**) respectively.

MSI analysis allowed to elucidate the biodistribution of the dosed drugs in the intestine specimens. Both MSI techniques allowed detection of all 4 dosed drugs. Whilst terfenadine, diphenhydramine and dextromethorphan were detected as [M+H]^+^ species (at *m*/*z* 472.32, 256.17 and 272.20 respectively) with either technique. Losartan was however detected as [M+H]^+^ species at *m*/*z* 423.17 by MALDI-MSI and as [M+K]^+^ species at *m*/*z* 461.129 by DESI-MSI. The control sections analyzed by DESI-MSI showed little interference through chemical background signals. Overall, the highest drug abundances were detected at 1 h post dose followed by a decline over time. The MALDI data followed the trend with the difference that Losartan had some chemical background for the selected mass window on control sections. A relatively low spatial resolution of 75 µm was chosen for the performed DESI-MSI experiments as it allowed to analyze each slide, holding 15 specimens, in an overnight experiment of approximately 12 h (Figure 2a).

The spatial resolution of the DESI-MSI experiments demonstrated that the majority of the drugs were in the mucosa/lumen and less in the submucosa/muscularis. However, it does not allow to clearly distinguish between mucosal villi and the intestinal lumen (Figure 2d). However, samples could be analyzed by MALDI-MSI with a spatial resolution of 10 µm at which the differentiation between the intestinal lumen and mucosal villi was made possible (Figure 2e).

**Figure 3:**
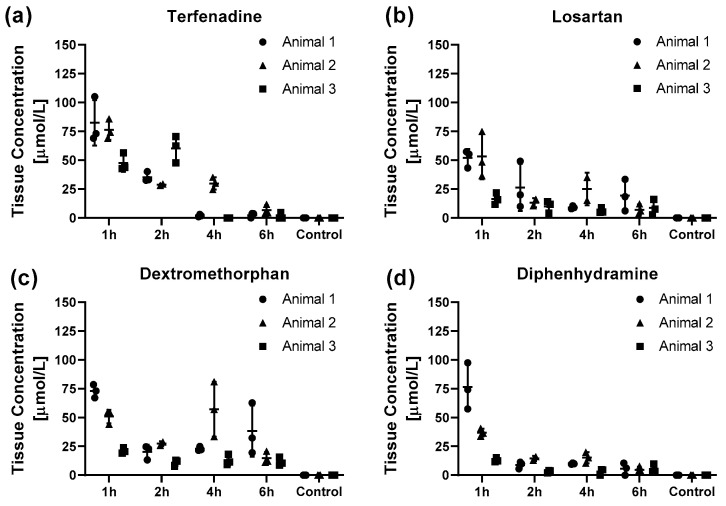
Pharmacokinetic profiles of the drug tissue concentrations for (**a**) terfenadine, (**b**) losartan, (**c**) dextromethorphan and (**d**) diphenhydramine for the different time points post-dosing obtained by LC-MS/MS analysis. The data is presented as individual marker for the technical replicates and mean ± SD of the animal. As the sample collection was terminal, animals 1–3 represent three different animals for each timepoint except for animal 2, 2 h post dose as one of the specimens was lost during the extraction process. One specimen from Animal 2, 4 h post dose was excluded as it exceeded the upper limit of the calibration range.

Even though the data is limited to total concentrations, to date LC-MS/MS quantification of tissue extracts remains the standard tool in drug pharmacodynamic studies (alongside quantitative whole body autoradiography) [22,23,24]. Therefore LC-MS/MS was performed to demonstrate the quantitative relationship in drug tissue concentrations and MSI results. In agreement with the imaging data, the absolute drug concentration for all four drugs showed overall a rapid decline from the 1 h to the 2 h post dose animals and a slower decline for the later timepoints (Figure 2). Terfenadine shows a slower decline with no remaining drug 4 h post dose in two out of three animals (Figure 2a). For all four drugs the absolute intra and inter animal concentrations showed little variation with only single specimens showing higher concentrations (Figure 3). Compared to the later time-points, the inter animal variability 1 h post dose was much larger with around 5-fold higher for diphenhydramine concentrations for animal 1 compared to animal 3 (Figure 3d).

**Figure 4:**
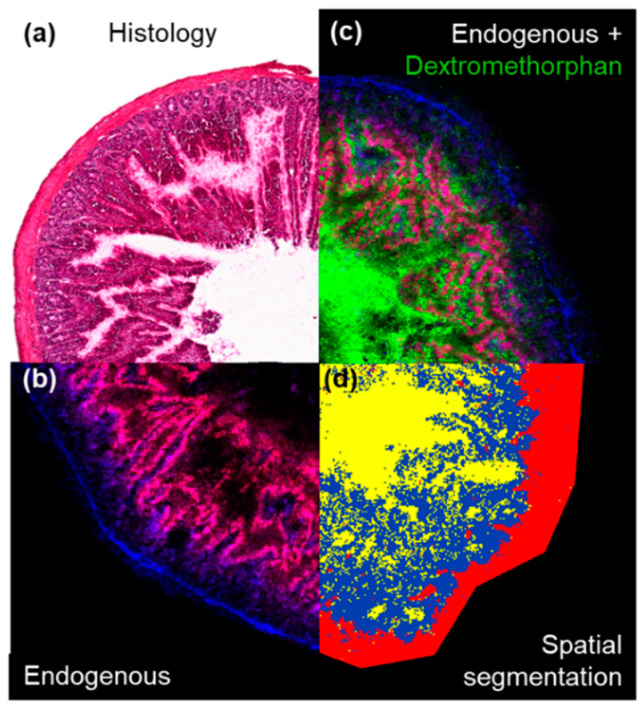
Extraction of the region-specific drug abundances in the different morphological features of the intestine: (**a**) The H&E stained tissue shows the typical morphology of a representative intestine section. (**b**) Shows the tissue outline determined by MALDI-MSI performed on an adjacent tissue section (endogenous lipids PC (34:2) [M+K]^+^ (*m*/*z* 796.53) (red) and PC (34:1) [M+K]^+^ (*m*/*z* 798.54) (blue). (**c**) Shows the relative localization of dextromethorphan (*m*/*z* 272.20) within the tissue. (**d**) Shows the result of the spatial segmentation bisecting K-means clustering of the tissue performed on features detected between *m*/*z* 750 to 850. Yellow = intestinal lumen, blue = mucosa and red = unresolved mixture of mucosal crypts, submucosa and muscularis respectively.

In contrast, MSI data provided a spatial dimension which could be manipulated and divided into the underlying histological tissue types [25,26]. Unsupervised, data-driven segmentation, performed on the lipid-containing mass range between *m*/*z* 750 and 850 allowed to “digitally dissect” the herein analysed intestinal sections into lumen, mucosa and an unresolved mixture of mucosal crypts, submucosa and muscularis as identified when compared to post-analysis haematoxylin and eosin (H&E) stained tissues (Figure 4). The abundance information for any detected analyte such as of endogenous metabolites or drugs could be extracted for each of the determined segments and subjected to statistical analysis or evaluation of the tissue concentration-time profiles of the drugs.

**Figure 5:**
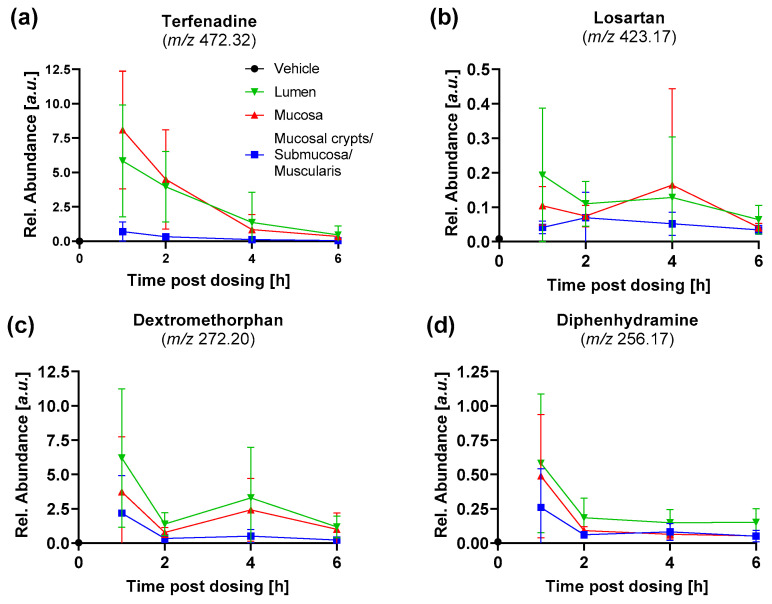
Abundance-time profiles of the four dosed drugs in the resolved tissue segments for (**a**) terfenadine, (**b**) losartan, (**c**) dextromethorphan and (**d**) diphenhydramine. Data is presented as mean ± SD of the nine intestinal segments analysed for each timepoint. The datapoint for the vehicle at T_0_ shows the mean background noise signal for the respective drug.

Extraction of the abundances of the four dosed drugs in the different segments of the intestine allow to compare tissue exposure-time profiles in the different morphological compartments of the intestine (Figure 5). The luminal tissue abundances follow a time profile as expected after oral dosing, with rapid initial uptake manifesting in the rapid decline between one and two hours post dosing and lower flux rates with decreasing luminal drug concentrations. Both, losartan and dextromethorphan show an plateau, or even increase, of the abundance in the lumen between two and four hours post dosing. This is likely the result of active excretion of the unmetabolized drugs into the bile and re-entering into the intestine as art of the enterohepatic recycling. The abundance-time profiles of the four drugs in the mucosa follow overall the drug abundances in the intestinal lumen. The high degree of correlation between these two compartments highlights the diffusion-driven, passive uptake of the drugs into the tissues. Interestingly, the tissue abundances in the outermost segment, which was comprised of mucosal crypts, submucosa and muscularis, showed overall similar profiles, albeit at much lower levels. The profiles show highest variability at datapoints closest to t_max_ which appears to be prior to the 1 h post dose datapoint for terfenadine, dextromethorphan and diphenhydramine. However, losartan was detected with highest abundance at 2 h post dose (Figure 5b).

The spatial information provided by the MSI analysis, could also be leveraged to estimate the drug fractions in the lumen and the absorbed drug fraction which was detected in the tissue (Table 1). This approach estimated the mean absorbed fraction of the drugs to range from 56% for terfenadine up to 66% for Losartan (Table 1). The high variability in the absorbed drug fraction was found to be primarily based on differences in the amount of drug-containing bowel content present in the specimens, depending on the thoroughness when removing the bowel content during necropsy. The interfering effect of the bowel content in the present study is analogous to the commonly observed contamination of tissues with blood, measuring drugs from the circulation [27,28] rather than pure tissue concentrations. Such interferences highlight the value of spatially resolved MSI experiments to enable precise localization of the drugs within even small morphological features of the tissue. The understanding of the drug distribution within the tissue can help to build better understanding of a compound’s PK properties and draw the correct conclusions from extract-based quantification approaches.

**Figure 6:**
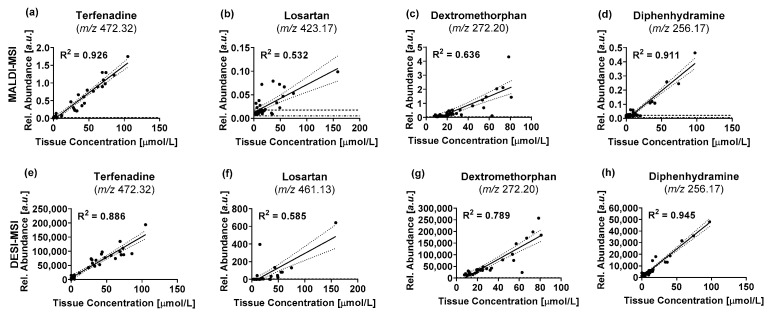
Comparison of drug tissue concentrations (terfenadine (**a**,**d**), losartan (**b**,**f**), dextromethorphan (**c**,**g**), diphenhydramine (**d**,**h**)) determined by LC-MS/MS and MALDI-MSI (**a**–**d**) and DESI-MSI (**e**–**h**), respectively. Each datapoint describes the measured data for one of the three intestinal segments that were analyzed for each animal. Relative abundances for MSI data are represented by the mean abundance of the whole tissue section. — Indicate best-fit linear regression lines … indicate the 95% confidence interval of the regression lines -·- indicate the LOD for the respective MSI technique --- indicate the LLOQ for the respective imaging technique.

A comparison was performed between the concentrations determined by LC-MS/MS and the mean tissue abundances determined by DESI-MSI and MALDI-MSI respectively to validate the visual agreement between the absolute concentrations and the ion images obtained by MSI (Figure 6). For the linear regression, the concentration of each specimen was compared to the mean tissue abundance of the corresponding section analyzed by MSI. The data for all timepoints and replicates was pooled for the comparison to include a wide range of tissue concentrations. The correlation of datasets obtained by using different techniques can give interesting insights into the underlying analytical phenomena. A positive shift of the *x*-axis intercept indicates a lower limit of detection (LOD) and lower limit of quantification (LLOQ) of the technique plotted on the *x*-axis, whilst a positive shift of the *y*-axis intercept indicates a lower LOD and LLOQ of the technique plotted on the *y*-axis. When using raw data without baseline removal, a positive shift of the intercept can be caused by unspecific background signals or chemical background as it is the case for the data reported below. This will be reflected in a shift of the LOD. The slope of the linear regression line will reflect the sensitivity of either method, with a 45° angle describing a “perfect” correlation in which both techniques have the same response factors. A deviation indicates different sensitivities for the compared techniques. The coefficients of determination (R^2^) describes the variance in the data explained by the correlation. It is effectively a measure for the deviation of the data points from the regression line and can indicate a deviation from a linear relationship or hint towards the presence of outlier in the data when the computed R^2^ values are low.

Overall, both techniques showed a comparable correlation between the drug abundances determined by MSI and the absolute drug concentration across all samples determined by LC-MS/MS. Both imaging techniques had a strong correlation for terfenadine (Figure 6a,e) and diphenhydramine (Figure 6d,h) with R^2^ values ranging between 0.886 and 0.945. Dextromethorphan showed a slightly lower correlation with R^2^ values of 0.636 for MALDI and 0.789 for DESI-MSI respectively (Figure 6c,g). Only losartan showed just a moderate correlation (R^2^ = 0.532 for MALDI and R^2^ = 0.585 for DESI respectively) due to the low sensitivity for the drug with either imaging technique (Figure 6b,f). The low sensitivity for losartan arose from a poor detection of the compound with either MSI technique, but particularly for MALDI-MSI where numerous data points fall between the LOD and LLOQ, which is reflected in the large variance of the data. The limited sensitivity for the drug was also reflected in the large 95% confidence interval (CI) of the regression lines for either technique, especially in the higher concentration range where a limited number of datapoints defined the regression line. The correlation between tissue concentrations and the MSI data proves a basis to use the MSI data for relative quantification of the drugs within and between specimens even without spray-depositing internal standard across the slide to compensate for local ion suppression effects. Some datapoints deviate substantially from the regression lines. These outliers were often inconsistent between the different benchmark compounds, even within the same tissue section, limiting the likelihood of these being systematic artefacts of sample preparation or analysis. Furthermore, the same pattern can be seen between the two imaging modalities, which provide independent data obtained from separate sections. Even though efforts were made to match the specimen fragments used for LC-MS/MS quantification and those used to generate thin sections for MSI analysis as much as possible, several millimeters can be between the analyzed specimens. We attributed some of the observed variation to differences in the analyte distribution in the intestines and thus analyzed specimen.

**Figure 7:**
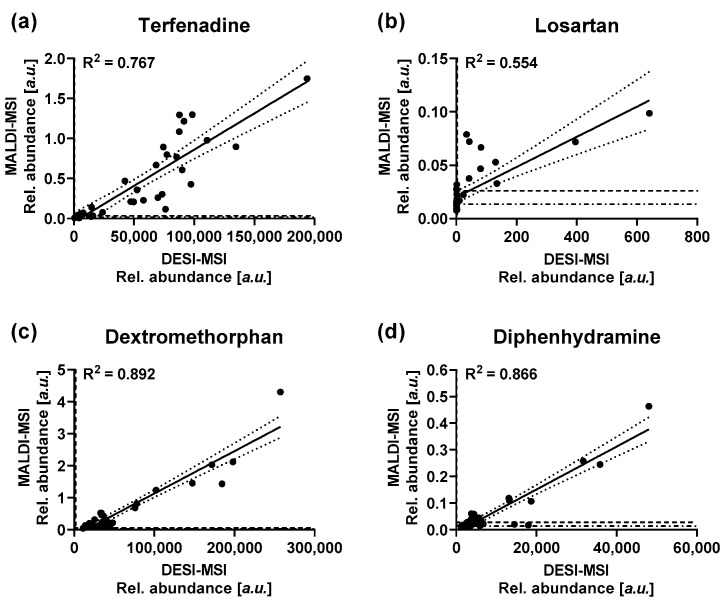
Correlation of the average drug abundances as detected by DESI-MSI and MALDI-MSI for (**a**) terfenadine, (**b**) Losartan, (**c**) dextromethorphan and (**d**) diphenhydramine. — Indicate best-fit linear regression lines … indicate the 95% confidence interval of the regression lines -·- indicate the LOD for the respective MSI technique --- indicate the LLOQ for the respective imaging technique.

A correlation analysis between the relative abundances for the four drugs was performed to further delineate differences in the data obtained with the two MSI techniques (Figure 7). The data obtained by the two MSI techniques is in good agreement for dextromethorphan and diphenhydramine with R^2^ values of 0.892 and 0.866 respectively. Dextromethorphan showed a noticeable right shift of the *x*-axis intercept from the origin, highlighting a lower LOD for the compound by MALDI-MSI (Figure 7). Terfenadine showed a slightly lower agreement between the two techniques with an R^2^ value of 0.767. As for the correlation of the drug tissue concentration and the imaging data, losartan showed the lowest agreement between the two MSI techniques with an R^2^ value of 0.554. As seen above, either MSI technique seemed to have a limited sensitivity for the drug under the analytical conditions. The noticeable positive shift of the y-intercept away from the origin indicating increased chemical background for the compound by MALDI-MSI (Figure 7b). Overall, both techniques showed comparable performance for the accuracy of the obtained data, with slight deviation based on preferential desorption/ionization under the specific analytical conditions dictated by the two techniques. The data obtained with either technique shows a sufficient correlation with the underlying tissue concentrations to justify “digital dissection” of the tissues and use of the extracted abundances for relative quantification of analytes without the use of an isotopically labelled standard, as long as the chosen spatial resolution allows to resolve the underlying morphological features in a tissue.

## 3. Discussion

We successfully co-embedded and prepared multiple intestine specimens which enabled simultaneous preparation of up to 15 specimens per mold, significantly reducing the time required to process the samples without interfering with a quantitative evaluation of the results. All samples analyzed in an experiment were prepared at the same time and under the same conditions, reducing the possibility of time-course effects due to analyte degradation/alteration observed when samples remain in the cryostat for extended periods of time. Preservation of the tissue morphology allowed to use the obtained tissue sections for high resolution MSI experiments and histological evaluation. Both, DESI-MSI and MALDI-MSI performed overall comparably good, albeit DESI-MSI had a tendency to show better sensitivity and lower limits of detection for the benchmarking compounds used in this work The correlation between MSI and absolute drug concentrations demonstrates minimal interference through the manipulation of the tissues during the embedding process and allows determination of accurate, quantitative results. The correlation also proves the basis for MSI-based relative quantification of the data after segmentation of the resulting distribution maps into the underlying morphological tissue compartments. Significantly, the correlation remains if the chemical composition of the different morphological compartments is not vastly different. This justifies the relative quantification of endogenous metabolites or of drug candidates during early development within and between comparable tissues without normalization to an isotopically labelled standard. However, fundamental changes in the chemical composition of tissues are likely to result in drastic changes in local ion suppression effects, distorting the elucidated biodistributions. This becomes particularly important when performing relative quantification across multiple tissue types or vastly different models, in which case the use of an isotopically labelled standard should be considered to ensure delivery of accurate results. The presented work also builds a foundation for a systematic evaluation of the changes in local ion suppression effects based on differences in the chemical composition of the tissues. Determination of intra- and inter-organ changes in ion suppression and consequences for the segmentation of biodistributions will be explored in future studies, including evaluation of normalization and compensation strategies.

## 4. Materials and Methods

### 4.1. Chemicals

Polyvinylpyrrolidone (PVP) (MW 360 kDa), (Hydroxypropyl)-methylcellulose (HPMC) (viscosity 40–60 cP, 2% in H_2_O (20 °C)), 2,5-dihydroxybenzoic acid (DHB), trifluoroacetic acid (TFA), terfenadine, terfenadine-*d3*, dextromethorphan hydrobromide, dextromethorphan-*d3*, diphenhydramine hydrochloride and diphenhydramine-*d3* were purchased from Merck (Darmstadt, Germany). Methanol, water, iso-pentane (2-Methyl-butane), isopropanol and acetonitrile (ACN) were obtained from Fisher Scientific (Waltham, MA, USA). Losartan-potassium salt was obtained from Cambridge Bioscience (Cambridge, UK). Losartan-*d4* was purchased from Toronto Research Chemicals (Toronto, ON, Canada). All solvents used were of analytical grade or higher.

### 4.2. Animals and Dosing

Adult male Han Wistar rats (approximate weight 260 g) were obtained from Charles River Laboratories (Margate, Kent, UK) and were acclimatized on site for a minimum of 3 days prior to dosing. Compounds were administered by oral gavage as cassette of terfenadine, losartan, diphenhydramine and dextromethorphan, formulated in 5% dimethyl sulfoxide/95% (30% *w*/*v* Captisol in water). Animals were dosed with 25 mg/kg/drug and euthanized at 1, 2, 4 or 6 h post-dosing. Vehicle control animals were euthanized at the latest sampling time at 6 h post dose. The first 20 cm of the small intestine were cut into four pieces each and snap frozen free-floating in dry ice chilled isopentane. For each animal, 3 intestine pieces were randomly selected for this study. The pieces were each split into half, one half was extracted for LC-MS/MS quantification, the other half was embedded and subject to MSI analysis. All tissue dissection was performed by trained AstraZeneca staff (project license 40/3484, procedure number 10).

### 4.3. Preparation of Intestine Specimens for MSI Analysis

The specimens were embedded and prepared according to a previously reported sample preparation workflow [21]. Briefly, the intestine specimens were co-embedded in a (Hydroxypropyl)-methylcellulose (HPMC) + Polyvinylpyrrolidone (PVP) hydrogel to enable time-efficient sectioning under comparable conditions for all specimens analyzed in one experiment. A total of 15 specimens was placed upright in peel-a-way molds (Thermo Scientific, Waltham, MA, USA) pre-filled with ice-cold embedding medium. Snap freezing of the filled mold was performed in dry ice-chilled isopropanol followed by a wash in dry ice chilled iso-pentane to wash off the excess of isopropanol. The frozen molds were kept on dry ice to allow evaporation of the adherent iso-pentane before they were sectioned on a cryostat. Sections of 10 µm thickness were cut at −18 °C and thaw mounted on either SuperFrost (Thermo Scientific, Waltham, MA, USA) or ITO coated (Bruker Daltonik, Bremen, Germany) slides for DESI or MALDI experiments respectively. To preserve the analyte integrity, samples were desiccated under nitrogen immediately after the thaw mounting, packed in vacuum-sealed slide mailer and stored in a −80 °C freezer until analysis [29].

### 4.4. DESI-MSI

Analysis was performed on a Q-Exactive mass spectrometer (Thermo Scientific, Bremen, Germany) equipped with an automated 2D-DESI ion source (Prosolia Inc., Indianapolis, IN, USA) operated in positive ion mode between *m*/*z* 100 to 900 with a nominal mass resolution of 70,000 at *m*/*z* 200. The injection time was fixed to 50 ms resulting in a scan rate of 3.7 pixel/s. A home-built DESI sprayer [30] was operated with a mixture of 95% methanol, 5% water delivered with a flow rate of 1.5 µL/min and nebulized with nitrogen at a backpressure of 6 bar. The spatial resolution was set to 75 µm. The resulting .raw files were converted into mzML files using ProteoWizard msConvert [31] (version 3.0.4043) and subsequently compiled to an .imzML file (imzML converter [32] version 1.3). All subsequent data processing was performed in SCiLS Lab (version 2019b, Bruker Daltonik, Bremen, Germany). The LOD and LLOQ for the four drugs were calculated 3× and 10× the standard deviation of the abundance of the respective mass filter of each drug across the vehicle tissues.

### 4.5. MALDI-MSI

Analysis was performed on a RapifleX Tissuetyper instrument (Bruker Daltonik, Bremen, Germany) operated in positive ion mode using 2,5-Dihydroxybenzoic acid (DHB) prepared in 50:50:0.1 ACN:water:TFA and spray deposited using an automated spray system (HTX technologies, Chapel Hill, NC, USA) following a previously reported protocol [33]. Drug distribution studies on intestines were performed with a spatial resolution of 10 µm in the mass range between *m*/*z* 200 and 1000. A total of 100 laser shots were summed up per pixel to give the final spectra. For all experiments the laser was operated with a repetition rate of 10 kHz. All raw data was directly uploaded and processed in FlexImaging (Bruker Daltonik, Bremen, Germany) or SCiLS lab software packages (Version 2019b). All reported MALDI data and images were normalized to the total ion current (TIC) to compensate for spectrum-to-spectrum variation [34]. The tissue segmentation for the estimation of the absorbed drug fraction were performed using bisecting k-means clustering using the build-in function in SCiLS lab with correlation distance as metric. The segmentation was performed using peaks in the mass range between *m*/*z* 750 and 850 which contains structural lipids outlining the morphological features of the intestine and the output maps visually compared to the underlying histology. The LOD and LLOQ for the four drugs were calculated 3× and 10× the standard deviation of the abundance of the respective mass filter of each drug across the vehicle tissues.

### 4.6. LC-MS/MS Sample Preparation and Analysis

Tissues were weighed into extraction tubes and 15 µL of mixed deuterium-labeled drugs prepared in acetonitrile (ACN) (1 µmol/L/drug) were added as internal standards into each tube. After addition of zirconia beads and 0.75 mL ice-cold ACN, tissues were homogenized using a tissue homogenizer (Precelllys 24, Bertin Technologies SAS, Montigny-le-Bretonneux, France). The homogenization was performed in 3 cycles, each consisted of 45 s of shaking at 4600 rpm followed by 30 s of pause and another 45 s of shaking. To allow samples to cool down, tubes were stored on ice for 15 min in between cycles. Samples were centrifuged at 14,000× *g* for 20 min under refrigeration (PrismR, Labnet international Inc., Edison, NJ, USA) and the supernatant was transferred into a new extraction tube. The residue was reconstituted in 0.75 mL ice-cold ACN followed by centrifugation for 20 min at 14,000× *g* under refrigeration. The supernatants for each sample were pooled. Aliquots of the supernatant were diluted 3-fold with HPLC-MS grade water and further diluted 1:10 in 25% ACN in water. Calibration standards were prepared from vehicle dosed control specimens as described above. Appropriate concentration of the drug standards prepared in ACN were added to the extraction tube before homogenizing the tissues. The calibration curves used for the drug quantification can be found in the Supplementary Information (Figure S1).

Chromatographic separation was performed on an UPLC system (Waters Corp., Milford, MA, USA) equipped with a BEH C18 column (Waters, Milford, MA, USA) with the following dimensions: 100 mm × 2.1 mm i.d. and a particle size of 1.7 μm. The column was operated at a temperature of 55 °C. 0.1% formic acid in water was used as aqueous mobile phase (A) whilst ACN was used as organic phase (B). The flow rate of the mobile phase was 0.5 mL/min throughout the gradient. 5 µL of each sample were injected and separated with the following gradient: T = 0 min 5% B, 0.5 min 5% B, 0.51 min 50% B, 4.5 min 80% B, 4.51 min 99% B till T = 5 min 99% B. The approximately 2 min lasting injection cycle of the LC system was used to re-equilibrate the column to 5% B for the next injection.

A Xevo TQ-XS (Waters, Wexford, IE) triple quadrupole instrument was used for mass spectrometric detection. Multiple Reaction Monitoring (MRM) transitions for all compounds were optimized on standards using IntelliStart and used as provided by the software (Table 2). The other instrument settings were used as followed: capillary voltage: 3.5 kV (+ve), nebulizing gas 1200 L/h, desolvation temperature 350 °C, cone gas 150 L/h.

## Figures and Tables

**Table 1:** The absorbed fraction of the four different drugs. The drug abundance of the tissue was extracted from the combined blue and red clusters seen in the spatial clustering. The data is presented as mean ± SD and range of all analyzed specimens. The individual values for each specimen can be found in Supplementary Figure S2.

	Absorbed Drug Fraction [%]		
Drug	Mean ± SD	Min	Max
Terfenadine	56 ± 23	8	89
Losartan	66 ± 24	12	93
Dextromethorphan	57 ± 22	10	92
Diphenhydramine	64 ± 21	18	93

**Table 2:** MRM transitions for the drugs and respective internal standards. The peak area of the quantifier trace was used for the quantification whilst the qualifier trace was used to verify the identity of the integrated peak.

	Quantifier Transition	Qualifier Transition
Diphenhydramine	256.3 > 167.0	256.3 > 165.0
Diphenhydramine-*d3*	259.3 > 167.0	259.3 > 165.1
Dextromethorphan	272.4 > 147.0	272.4 > 215.1
Dextromethorphan-*d3*	275.4 > 215.1	275.4 > 147.0
Losartan	423.5 > 207.0	423.5 > 179.9
Losartan-*d4*	427.5 > 211.1	427.5 > 184.0
Terfenadine	472.1 > 91.0	472.7 > 436.3
Terfenadine-*d3*	475.7 > 91.0	475.7 > 438.4

## Data Availability

All relevant data is represented in the manuscript or the supplementary materials. The MSI raw data was deposited in the Imperial College London Research Data Repository under the DOI:10.14469/hpc/10255.

## Supplementary Materials

The following are available online at https://www.mdpi.com/article/10.3390/metabo12030261/s1, Figure S1: Linear regression lines for the internal standard (IS) normalized peak areas over the standard concentration; Figure S2: Individual values for the estimated absorbed drug fractions.
