# Supplementary material for: Correlating Mass Spectrometry Imaging and Liquid Chromatography-Tandem Mass Spectrometry for Tissue-Based Pharmacokinetic Studies
Source: Metabolites. 2022 Mar 18;12(3):261. doi: 10.3390/metabo12030261 (PMC8954739; doi:10.3390/metabo12030261)
Supplement: Supplementary file 1 [file metabolites-12-00261-s001.zip › metabolites-1618410-supplementary.pdf]

Supplementary information: “Correlating mass spectrometry imaging and liquid chromatography-tandem mass spectrometry for tissue-based pharmacokinetic studies”

Andreas Dannhorn[1,2], Emine Kazanc[1], Gregory Hamm[2], John Swales[2], Nicole Strittmatter[2], Gareth Maglennon[2], Richard JA Goodwin[2], Zoltan Takats[1,5,6*]

1 Department of Metabolism, Digestion and Reproduction, Imperial College London, London, UK

2 Imaging & Data Analytics, Clinical Pharmacology and Safety Sciences, R&D, AstraZeneca, Cambridge, UK

3 Oncology Safety, Clinical Pharmacology and Safety Sciences, R&D, AstraZeneca, Cambridge, UK

4 Institute of Infection, Immunity and Inflammation, College of Medical, Veterinary and Life Sciences, University of Glasgow, Glasgow, UK

5 Laboratoire PRISM Inserm U1192 - University of Lille,Villeneuve d’Ascq ,France

6 The Rosalind Franklin Institute, Harwell, UK

Supplementary Figure S1:

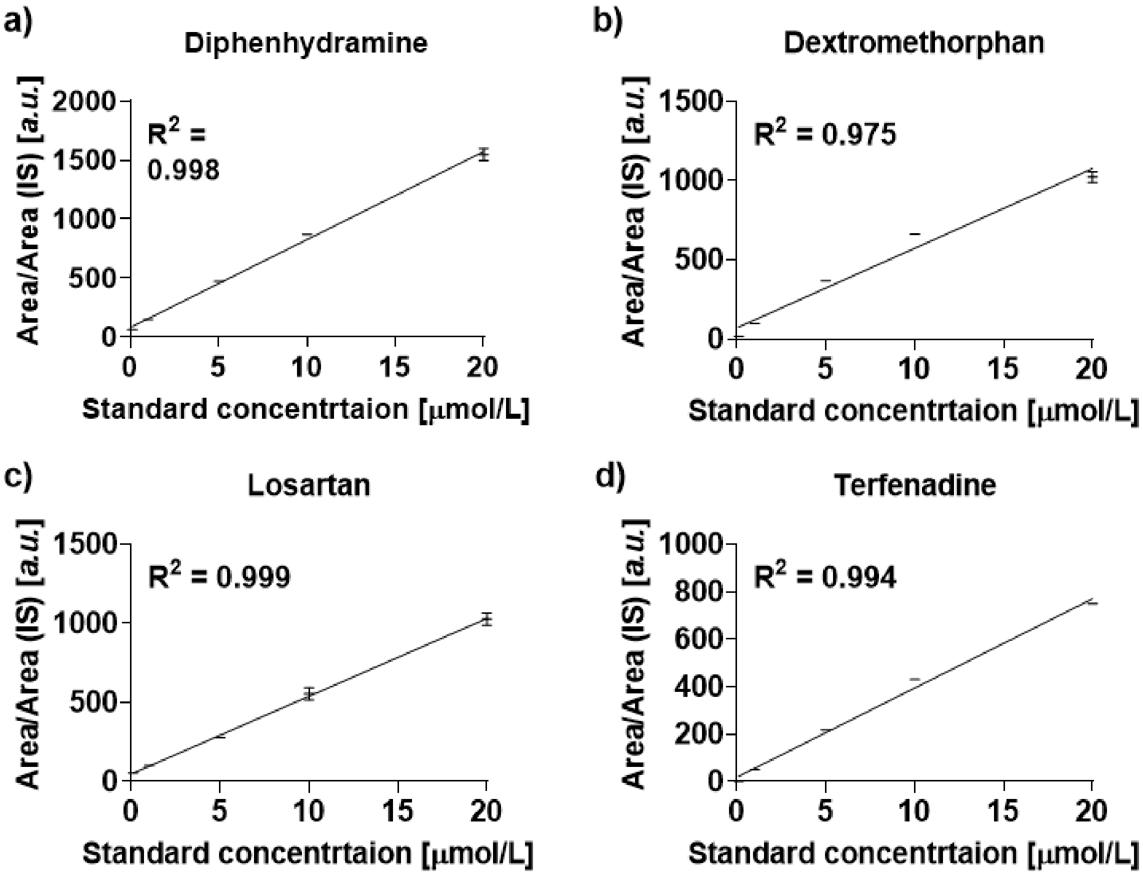

Figure S1. Linear regression lines for the internal standard (IS) normalized peak areas over the standard concentration: for (**a**) diphenhydramine, (**b**) dextromethorphan, (**c**) losartan and (**d**) terfenadine. Data is presented as mean ± SD, *n* = 4 replicates per measurement

Supplementary Figure S2:

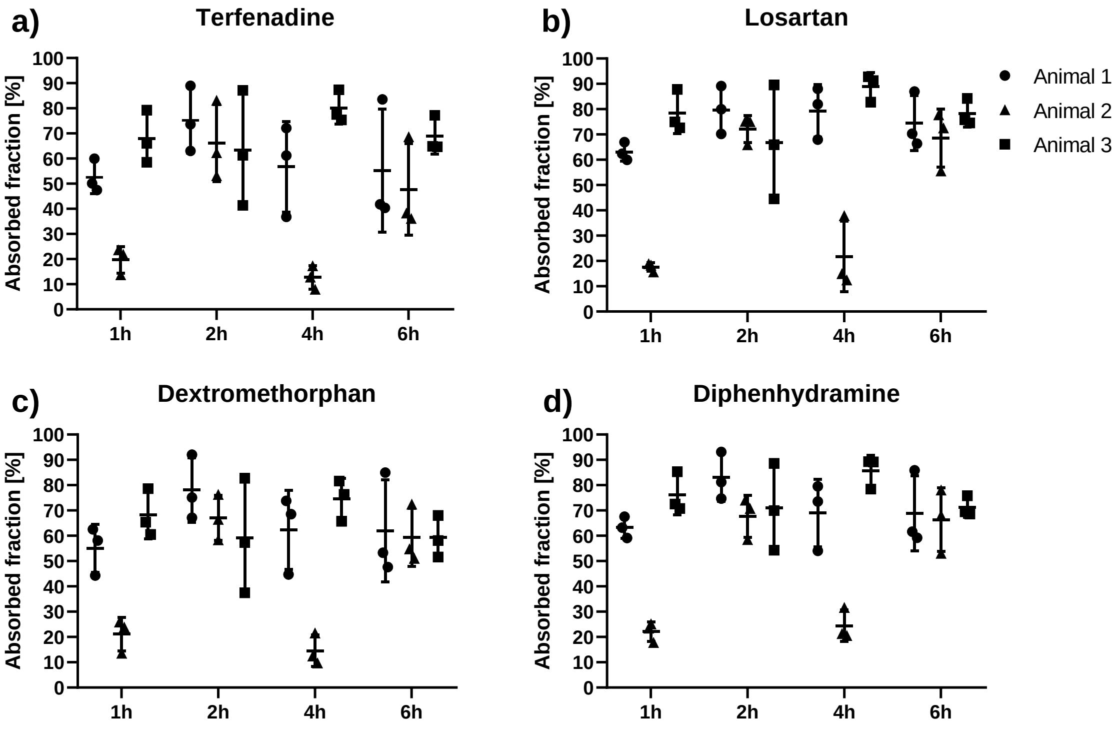

Figure S2. Individual values for the estimated absorbed drug fractions: for (**a**) terfenadine, (**b**) Losartan, (**c**) Dextromethorphan and (**d**) diphenhydramine. As the sample collection was terminal, animals 1-3 represent different animals for each timepoint. The assignment of the symbols is identical with the assignment in Figure 2.
